# Control of Risk Factors Among People With Diagnosed Diabetes, by Lower Extremity Disease Status

**Published:** 2009-09-15

**Authors:** Rashida R. Dorsey, Mark S. Eberhardt, Edward W. Gregg, Linda S. Geiss

**Affiliations:** Dr Dorsey is affiliated with the Epidemic Intelligence Service and the National Center for Health Statistics, Centers for Disease Control and Prevention (CDC); National Center for Health Statistics, Centers for Disease Control and Prevention (CDC), Hyattsville, Maryland; Division of Diabetes Translation, CDC, Atlanta, Georgia; Division of Diabetes Translation, CDC, Atlanta, Georgia

## Abstract

**Introduction:**

We examined the control of modifiable risk factors among a national sample of diabetic people with and without lower extremity disease (LED).

**Methods:**

The sample from the 1999-2004 National Health and Nutrition Examination Survey consisted of 948 adults aged 40 years or older with diagnosed diabetes and who had been assessed for LED. LED was defined as peripheral arterial disease (ankle-brachial index <0.9), peripheral neuropathy (≥1 insensate area), or presence of foot ulcer. Good control of modifiable risk factors, based on American Diabetes Association recommendations, included being a nonsmoker and having the following measurements: HbA1c less than 7%, systolic blood pressure less than or equal to 130 mm Hg, diastolic blood pressure less than or equal to 80 mm Hg, high density lipoprotein (HDL) cholesterol greater than 50 mg/dL, and body mass index (BMI) between 18.5 kg/m^2^ and 24.9 kg/m^2^.

**Results:**

Diabetic people with LED were less likely than were people without LED to have recommended levels of HbA1c (39.3% vs 53.5%) and HDL cholesterol (29.7% vs 41.1%), but there were no differences in systolic or diastolic blood pressure, BMI classification, or smoking status between people with and without LED. Control of some risk factors differed among population subgroups. Notably, among diabetic people with LED, non-Hispanic blacks were more likely to have improper control of HbA1c (adjusted odds ratio [AOR] = 2.0; 95% confidence interval [CI], 1.1-3.9), systolic blood pressure (AOR = 1.9; 95% CI, 1.1-3.2), and diastolic blood pressure (AOR = 2.6; 95% CI, 1.1-5.8), compared with non-Hispanic whites.

**Conclusion:**

Control of 2 of 6 modifiable risk factors was worse in diabetic adults with LED compared with diabetic adults without LED. Among diabetic people with LED, non-Hispanic blacks had worse control of 3 of 6 risk factors compared with non-Hispanic whites.

## Introduction

People who have diabetes are at an increased risk for developing lower extremity disease (LED), which includes peripheral arterial disease (PAD) and peripheral neuropathy (PN) (1). Both conditions are associated with mobility limitations, disability, diminished quality of life and increased medical expenditures (2). PAD coupled with PN may lead to foot ulceration and ultimately nontraumatic amputation if not treated effectively (1). In fact, diabetic people with PN or PAD have been shown to be 3 times more likely to have an amputation compared with diabetic people without these conditions, and the 5-year survival rate after a diabetes-related amputation has been estimated to be less than 30% (2,3).

People with LED are at increased risk for coronary heart disease and stroke. Prospective studies have shown PAD, measured by the ankle-brachial index (ABI), predicts subsequent myocardial infarction and cardiovascular mortality (4,5). An association has also been demonstrated between PN and cardiovascular disease among people with diabetes (6).

For diabetic adults with LED, improved control of modifiable risk factors may reduce the risk of progression to late-stage disease, which is responsible for the majority of health care expenditures, morbidity, mortality, and disability among people with diabetes. Lack of tightly controlled blood glucose, blood pressure, and cholesterol can put people with diabetes at an increased risk for long-term complications (7,8). Furthermore, improvements in blood pressure and lipid control are associated with a reduction in adverse cardiovascular events among people with PAD, and reductions in blood glucose can reduce progression to amputation among diabetic people with PN (7,9). However, clinical studies have shown suboptimal management of modifiable risk factors among people with LED (10).

Evaluating the associations between LED and modifiable risk factors among diabetic people with LED may identify intervention opportunities to prevent late-stage disease. Our objectives were to examine a nationally representative sample of people with diagnosed diabetes to 1) describe the prevalence of modifiable risk factors for people with and without LED, and 2) identify factors specifically associated with poor control of modifiable risk factors, stratified by LED presence or absence.

## Methods

### Data source

The National Health and Nutrition Examination Survey (NHANES) is a national population-based survey designed to assess the health and nutritional status of civilian, noninstitutionalized people older than age 2 months in the United States (11). The survey consists of an in-home interview in which participants are queried about health behaviors, health status, and risk factors and includes a physical examination conducted at a mobile examination center. Beginning in 1999, NHANES has been conducted continuously. We used data from NHANES 1999-2004 in this analysis. Only people aged 40 years or older with diagnosed diabetes were included (n = 1,160). People with diabetes were defined as those with a self-report of physician-diagnosed diabetes.

People aged 40 years or older who completed both the in-person interview and medical examination were assessed for the presence of LED, which included tests for PAD and PN, as well as physical inspection for foot lesions and abnormalities. Among people aged 40 years or older with diagnosed diabetes, 15 people with lower extremity amputations were excluded from analyses (1 non-Hispanic white, 5 non-Hispanic blacks, and 9 Mexican Americans). Participants with an ABI at or above 1.5 in both legs or in 1 leg if the ABI measurement for the opposite leg was missing were excluded because they may have medial arterial perfusion, which prevents accurate ABI measurement. Data on PAD for both feet were missing for 176 participants with diagnosed diabetes (15.2%), and data on PN for both feet were missing for 55 participants with diagnosed diabetes (2.9%) because of equipment failure, participant refusal, time constraints for physical examination, inability of respondent to undergo LED assessment, or other unspecified cause. Thus, 984 people were assessed for PAD, 1,105 were assessed for PN, and of these 948 had an assessment for both PAD and PN (83.1% of adults aged ≥40 with diagnosed diabetes).

PAD was assessed using the ABI, which is the ratio of systolic blood pressure in the ankles (posterior tibial vessels) to that in the right arm (right brachial vessel) (Parks Mini-Lab IV, Model 3100, Parks Medical Electronics, Inc, Aloha, Oregon) (12,13). Participants aged 40 to 59 years had 2 blood pressure measurements taken at the right arm and 2 measurements taken at both the left and right ankles. Left and right ABI were calculated as the mean of the 2 measurements. People aged 60 years and older were only given 1 blood pressure measurement per site, and this single measurement was used to calculate left and right ABI. People with any ABI measurement (left or right) <0.9 were considered to have PAD.

PN was assessed by testing foot sensation with the Semmes Weinstein monofilament (5.07-gauge nylon Semmes Weinstein monofilament, Mid-Delta Health Systems, Inc, Belzoni, Mississippi). Pressure was applied to each foot with the monofilament at 3 sites (plantar: first metatarsal head, fifth metatarsal head, and hallux). The monofilament was applied at each site until it buckled and then held for 1 second. A random forced-response method was used to determine whether the participant could feel the monofilament. A site was considered insensate if a person incorrectly identified when the monofilament was placed on that area of the foot for 2 of 3 applications. Participants with 1 or more insensate area (0-6 possible) were considered to have PN. Research has shown 1 or more insensate area to be predictive of amputation and foot ulcers, with strong specificity and sensitivity (14).

During the physical examination, participants' feet were visually examined by health technicians for any abnormalities. Participants with foot ulcers were also considered to have LED. Individuals with amputations (toe or foot; 1 leg or both legs) were excluded from analyses (n = 15) because they were considered to already have late-stage disease, and the purpose of this study is to examine prevalence of modifiable risk factors in earlier stages of disease (ie, PN and PAD).

Race and ethnicity were self-reported. We restricted race/ethnicity-specific analyses to non-Hispanic white, non-Hispanic black, and Mexican Americans due to limited sample size of other races. Covariates investigated include age, health insurance status, sex, and education level. Education was categorized as high school education or less, including certificate of general education development (GED), or some college or greater.

To evaluate control of risk factors among diabetic people with LED, the American Diabetes Association (ADA) Standards of Medical Care in Diabetes recommendations were used to establish variables that indicate levels of diabetic control (15), including glycemic control (hemoglobin A1c [HbA1c] <7% indicative of good control) and blood pressure control (systolic blood pressure ≤130 mm Hg and diastolic blood pressure ≤80 mm Hg indicative of good control). Smoking is a known risk factor for LED, and the ADA recommends not smoking as a part of good disease management (15,16). Participants were classified as never, former, or current smokers. Never smokers were defined as people who reported to not be currently smoking and to have smoked fewer than 100 cigarettes during their lifetime. Blood lipid control was assessed by measuring high-density lipoprotein (HDL) cholesterol. The ADA considers HDL cholesterol greater than 50 mg/dL as the low-risk lipid level for adults. (Low-density lipoprotein cholesterol [LDL] is the primary ADA-recommended blood lipid for assessment of blood lipid control and treatment. However, due to sample size limitations during the study period, LDL measures were not available for these analyses, so HDL cholesterol was used.) Weight level was investigated as well, as this has been associated with some forms of LED among people with diabetes as well as cardiovascular disease risk (15,17). Weight level was assessed by using body mass index (BMI) (18.5-24.9 kg/m^2^ = healthy body weight; 25.0-29.9 kg/m^2^ = overweight; ≥30.0 kg/m^2^ = obese). The ADA recommends weight loss for all overweight and obese people with diabetes and considers it a therapeutic objective (15).

### Statistical analyses

All estimates were weighted using NHANES sample weights. SAS version 9.1.3 (SAS Institute Inc, Cary, North Carolina) was used for data management, and SUDAAN version 9 (RTI International, Research Triangle Park, North Carolina) was used to account for the complex sampling scheme. *P* values <.05 were considered statistically significant, with no adjustment for multiple comparisons. We calculated the prevalence of modifiable risk factors by LED status and tested for significant differences using χ^2^ tests. Prevalence estimates were age-standardized to the 2000 US population, using the following age groups: 40-49 years, 50-64 years, and 65 years and older. To identify factors associated with poor control of modifiable risk factors, logistic regressions stratified by absence or presence of LED were used to model the odds of poor control for each modifiable risk factor (ie, HbA1c, systolic blood pressure, diastolic blood pressure, HDL cholesterol, BMI, and smoking status). Variables included in the models were age, sex, race/ethnicity, education level, and health insurance status. Adjusted odds ratios (AORs) and confidence intervals (CIs) were used to determine significance. If the CI did not include 1, the associated AOR was considered significant. For logistic modeling, obese and overweight categories were grouped and compared with normal weight, and never and former smokers were grouped and compared with current smokers. Two-way interactions were examined for sex and race/ethnicity; education level and race/ethnicity; and sex and education level.

## Results

Of 948 eligible participants, 365 had LED. Data on LED were missing for 212 diabetic people aged 40 years or older. Compared with respondents without missing LED data, those with missing LED information were more likely to have less than a high school education (46.6% vs 29.8%, *P* < .001), more likely to be female (61.1% vs 47.8%, *P* = .02), and more likely to be older than age 70 (56.0% vs 42.1%, *P* < .001) (data not shown).

Approximately 35% of diabetic people aged 40 years or older had LED. Among people with diabetes, 33.4% of non-Hispanic whites, 38.9% of non-Hispanic blacks and 31.9% of Mexican Americans had LED (Table 1).

The prevalence of 4 of 6 risk factors did not differ significantly between those with and without LED: systolic blood pressure at or above 130 mm Hg, diastolic blood pressure at or above 80 mm Hg, obese BMI, and current smoking (Table 2). However, the prevalence of 2 risk factors was significantly higher in people with LED than in those without LED: HbA1c of 7% or higher (60.7% vs 46.5%) and HDL cholesterol at or below 50 mg/dL (70.3% vs 58.9%).

Among diabetic adults with LED, non-Hispanic blacks were significantly more likely than non-Hispanic whites to have HbA1c levels at or above 7%, systolic blood pressure values at or above 130 mm Hg, and diastolic blood pressure values at or above 80 mm Hg (Table 3). No significant differences were found between non-Hispanic blacks and non-Hispanic whites with LED for HDL cholesterol control, smoking status, or weight level. No differences in diabetic control were found between non-Hispanic whites and Mexican Americans with LED.

Racial/ethnic differences in diabetes control were also observed among diabetic people without LED. Non-Hispanic blacks without LED were more likely to have uncontrolled diastolic blood pressure control (AOR = 2.2; 95% CI, 1.0-4.5) compared with non-Hispanic whites. Among those without LED, Mexican Americans were more likely to have HbA1c at or above 7% (AOR = 2.7; 95% CI, 1.8-4.1) compared with non-Hispanic whites. No significant associations were found between race/ethnicity and systolic blood pressure, HDL cholesterol, smoking behavior, or weight control for respondents without LED.

Sex and education level differences in risk factor control according to LED status were also observed in logistic modeling. Among people with and without LED, men were more likely to have HDL cholesterol at or below 50 mg/dL after adjusting for age, race/ethnicity, health insurance status, and education level (Table 3). Compared with people with some college education or greater, people without LED who had less than a high school education were more likely to have poor HDL and systolic blood pressure control. Among participants with LED, people who had less than a high school education were more likely than those with some college education or greater to be current smokers.

## Discussion

Approximately one-third of participants aged 40 years or older with diabetes had LED. A similar prevalence of risk factor control at the ADA-recommended level was observed in participants with and without LED for 4 of 6 measures (systolic blood pressure, diastolic blood pressure, BMI, and smoking status). People with LED had a significantly lower prevalence of risk factor control at the ADA recommended level compared with those without LED in 2 of 6 measures (HbA1c and HDL cholesterol).

In our study, people with LED were less likely to have tight glycemic and lipid control than were those without LED. Although incomplete success controlling these risk factors was observed in both groups, the implications may be worse for people with LED because they are at increased risk for cardiovascular events and amputation. Poor glycemic control has been associated with increased susceptibility to foot infection and poor wound healing among people with diabetes (18,19), and poor lipid control and poor glycemic control have been associated with cardiovascular mortality (20). Therefore, efforts to improve glycemic control of blood lipids among diabetic people with early stage LED may be an effective strategy in reducing subsequent amputations and cardiovascular events.

Among participants with LED, we found racial/ethnic differences in the level of a few risk factors that could influence progression to amputation and other cardiovascular outcomes. Non-Hispanic blacks were less likely to have adequate glycemic control and blood pressure control than were non-Hispanic whites, but no differences were observed for HDL control. Unlike non-Hispanic blacks, Mexican Americans had no differences in level of risk factor control among people with LED compared with non-Hispanic whites.

Racial/ethnic differences in late-stage disease have been observed. Data from the National Hospital Discharge Survey have shown that whites with diabetes have a lower rate of nontraumatic amputations than do blacks with diabetes (21). Additionally, higher rates of lower limb amputation have been shown to be directly attributable to peripheral vascular disease among blacks compared with whites (22). Differences in disease control may play a role in the racial/ethnic differences in amputation rates (23,24). Racial/ethnic differences in cardiovascular disease have also been documented. Compared with non-Hispanic whites with diabetes, non-Hispanic blacks with diabetes have higher rates of hospitalizations for cardiovascular events (25). Racial/ethnic differences observed in risk factor control may be associated with subsequent racial/ethnic differences in late-stage disease. Although the findings from our study do not provide direct support for that association, they do suggest that future studies aimed at clarifying potential racial/ethnic differences in diabetes control may be warranted.

Low education level was associated with an increased risk for poor control of modifiable risk factors among people with and without LED. This finding is similar to those of previous reports that have documented a higher prevalence of cardiovascular disease risk factors in people with less than high school education compared with people with some college education or greater (26,27).

Our findings have implications for national diabetes objectives. *Healthy People 2010* has established objectives to reduce the rate of lower extremity amputations in people with diabetes and to reduce deaths from cardiovascular disease in people with diabetes (28). Interventions focused on risk factor reduction among diabetic people, especially those with LED, may contribute to reducing lower extremity amputations and cardiovascular disease and help achieve *Healthy People 2010* objectives. Our findings also reinforce the importance of the Control your Diabetes for Life national education message promoted by the National Diabetes Education Program, American Diabetes Association, and American College of Cardiology, which encourages diabetes patients to maintain recommended levels of HbA1c, blood pressure, and cholesterol (29).

Our study has several limitations. First, only noninvasive tests were used to measure PAD and PN. Although more comprehensive tests may provide more accurate diagnostic testing, both the ABI test for PAD and the monofilament test for PN have been shown to have high degrees of both sensitivity and specificity (13). Additionally, the ADA recommends annual foot examinations that include PN testing with the monofilament and PAD testing with the ABI, and cutpoints for PAD and PN determination were based on clinical practice guidelines and epidemiologic studies (13-15). Second, we used HDL cholesterol to assess blood lipid control, but LDL cholesterol is the current preferred measure for assessment of blood lipid control and treatment. Third, physical activity level is associated with cardiovascular disease, but we did not analyze it as a risk factor because clinical recommendations for physical activity by diabetic people with LED have not been established (15). Furthermore, it is not known whether physical activity (especially weight-bearing activity) is contraindicated and therefore not promoted by health care providers for diabetic people with severe LED. Fourth, medication data were not used in these analyses. It was difficult to assess whether medications were appropriate for respondents' diabetic management needs, so interpreting these findings would be problematic. Fifth, data on LED were missing for approximately 12% of the sampled people aged 40 or older with diabetes. People with missing PAD data were more likely to be female, older, and have less than a high school education. These differences may have biased our results because increasing age and lower socioeconomic status have been linked to worse diabetes outcomes. Finally, NHANES does not include institutionalized people (eg, nursing home residents), and findings that include institutionalized people may differ from ours.

Our study provides the first estimates of modifiable risk factors among diabetic people with and without LED using nationally representative data. Our data show a higher prevalence of 2 of 6 modifiable risk factors studied that may affect progression to late-stage disease among diabetic adults with LED compared with those without LED. Our findings also show different levels of risk factor control for some risk factors among racial/ethnic groups with diabetes and LED. Exploring the potential role of risk factor control among people with LED may reduce amputations and cardiovascular events and may help explain and possibly reduce the observed racial/ethnic disparity in amputations.

## Figures and Tables

**Table 1:** Characteristics of People With Diagnosed Diabetes Aged 40 Years or Older, By LED Status, National Health and Nutrition Examination Survey, 1999-2004^a^

**Characteristic**	LED Present (N = 365)	LED Absent (N = 583)	*P* Value^b^
**Age, mean (SE)**	63.8 (0.92)	59.0 (0.54)	<.001
**Sex^c^, % (95% CI)**			
Male	64.6 (55.4-72.8)	48.2 (43.2-53.2)	<.001
Female	35.4 (27.2-44.6)	51.8 (46.8-56.8)	
**Education^c^, % (95% CI)**			
Less than high school education	30.7 (21.9-41.2)	25.5 (21.8-29.7)	.10
High school or GED	32.1 (23.7-41.8)	23.7 (19.1-29.0)	
Some college or greater	37.2 (28.0-47.6)	50.8 (45.2-56.3)	
**Health insurance, % (95% CI)**			
Present	93.4 (88.8-96.2)	88.5 (84.9-91.3)	.40
Absent	6.6 (3.8-11.2)	11.5 (8.7-15.1)	
**Race/ethnicity^c^, % (95% CI)**			
Non-Hispanic white	72.7 (62.9-80.7)	74.5 (67.4-80.5)	.25
Non-Hispanic black	18.5 (12.5-26.6)	15.7 (11.5-26.6)	
Mexican American	8.8 (5.2-14.5)	9.8 (6.4-14.8)	

Abbreviations: LED, lower extremity disease; SE, standard error; CI, confidence interval; GED, general education development certificate.

a Weighted estimates.

b *P* values derived from χ^2^ test.

c Age-standardized to the 2000 US population.

**Table 2:** Prevalence of Modifiable Risk Factors for People With Diagnosed Diabetes Aged 40 Years or Older, by LED Status, National Health and Nutrition Examination Survey, 1999-2004^a^

**Modifiable Risk Factors**	Sample Size^b^(N = 948)	LED Present (N = 365), % (95% CI)	LED Absent (N = 583), % (95% CI)	*P* Value^c^
**HbA1c, %**				
<7	458	39.3 (31.5-47.8)	53.5 (48.2-58.6)	.03
≥7	458	60.7 (52.3-68.5)	46.5 (41.4-51.8)	
**Systolic blood pressure, mm Hg**				
<130	405	51.8 (42.2-61.3)	49.2 (42.3-57.8)	.30
≥130	514	48.2 (38.7-56.2)	50.8 (43.8-57.7)	
**Diastolic blood pressure, mm Hg**				
<80	740	73.5 (64.0-81.3)	74.2 (68.5-79.2)	.80
≥80	179	26.5 (18.7-36.0)	25.8 (20.8-31.5)	
**HDL cholesterol, mg/dL**				
>50	356	29.7 (20.3-41.2)	41.1 (34.5-48.0)	.04
≤50	560	70.3 (58.8-79.7)	58.9 (52.0-65.5)	
**BMI classification^d^**				
Healthy	149	12.7 (7.3-21.0)	17.6 (13.2-23.0)	.09
Overweight	351	29.2 (21.1-38.9)	31.7 (25.2-38.9)	
Obese	430	58.1 (48.3-67.4)	50.7 (42.9-58.6)	
**Smoking status^d^**				
Never	431	40.9 (33.5-48.8)	47.7 (41.0-54.5)	.43
Former	357	33.8 (27.0-41.3)	32.8 (28.0-37.9)	
Current	160	25.3 (16.8-36.1)	19.5 (16.3-24.1)	

Abbreviations: LED, lower extremity disease; CI, confidence interval; HbA1c, hemoglobin A1c; HDL, high-density lipoprotein; BMI, body mass index.

a Age-standardized to the 2000 US population; percentages are weighted.

b Some cell sample sizes may not add to 948 because of missing data.

c *P* values derived from χ^2^ test.

d See Methods section for subgroup definitions.

**Table 3:** Odds of Modifiable Risk Factors for Adults With Diagnosed Diabetes, by LED Status, National Health and Nutrition Examination Survey, 1999-2004

**Modifiable Risk Factor**	**LED Present, AOR** a ** (95% CI)^b^**	**LED Absent, AOR** a ** (95% CI) b**
**HbA1c ≥7%**		
**Sex**		
Female	1 [Reference]	1 [Reference]
Male	0.9 (0.5-1.8)	0.8 (0.5-1.3)
**Race/ethnicity**		
Non-Hispanic white	1 [Reference]	1 [Reference]
Non-Hispanic black	2.0 (1.1-3.9)	1.6 (1.0-2.7)
Mexican American	1.1 (0.5-2.5)	2.7 (1.8-4.1)
**Education**		
Some college or greater	1 [Reference]	1 [Reference]
Less than high school	1.5 (0.6-3.9)	1.3 (0.7-2.4)
High school graduate or GED	1.3 (0.5-3.1)	0.9 (0.5-1.9)
**Health insurance coverage**		
Absent	1 [Reference]	1 [Reference]
Present	0.8 (0.3-2.3)	0.9 (0.3-2.4)
**Systolic blood pressure ≥130 mm Hg**		
**Sex**		
Female	1 [Reference]	1 [Reference]
Male	0.6 (0.3-1.1)	0.8 (0.5-1.3)
**Race/ethnicity**		
Non-Hispanic white	1 [Reference]	1 [Reference]
Non-Hispanic black	1.9 (1.1-3.2)	1.3 (0.8-2.3)
Mexican American	0.9 (0.5-1.9)	0.8 (0.4-1.6)
**Education**		
Some college or greater	1 [Reference]	1 [Reference]
Less than high school	1.6 (0.7-3.7)	1.9 (1.1-3.1)
High school graduate or GED	0.8 (0.3-1.7)	1.7 (0.8-3.5)
**Health insurance coverage**		
Absent	1 [Reference]	1 [Reference]
Present	1.5 (0.5-4.3)	1.3 (0.6-2.8)
**Diastolic Blood Pressure ≥80 mm Hg**		
**Sex**		
Female	1 [Reference]	1 [Reference]
Male	1.8 (0.7-4.3)	1.5 (0.8-3.0)
**Race/ethnicity**		
Non-Hispanic white	1 [Reference]	1 [Reference]
Non-Hispanic black	2.6 (1.1-5.8)	2.2 (1.0-4.5)
Mexican American	0.4 (0.1-1.8)	1.2 (0.5-2.8)
**Education**		
Some college or greater	1 [Reference]	1 [Reference]
Less than high school	1.4 (0.5-4.1)	1.7 (0.9-3.3)
High school graduate or GED	0.4 (0.1-1.3)	1.6 (0.7-3.9)
**Health insurance coverage**		
Absent	1 [Reference]	1 [Reference]
Present	0.2 (0.1-1.1)	0.5 (0.2-1.1)
**HDL ≤50 mg/dL**		
**Sex**		
Female	1 [Reference]	1 [Reference]
Male	3.2 (1.4-7.4)	2.7 (1.4-5.3)
**Race/ethnicity**		
Non-Hispanic white	1 [Reference]	1 [Reference]
Non-Hispanic black	0.4 (0.1-1.3)	1.0 (0.5-1.9)
Mexican American	0.6 (0.2-1.9)	0.7 (0.2-2.0)
**Education**		
Some college or greater	1 [Reference]	1 [Reference]
Less than high school	0.8 (0.3-2.2)	2.0(1.1-3.9)
High school graduate or GED	0.3 (0.1-1.3)	1.4 (0.7-2.9)
**Health insurance coverage**		
Absent	1 [Reference]	1 [Reference]
Present	0.2 (0.1-0.9)	2.4 (0.7-8.1)
**Current smoker**		
**Sex**		
Female	1 [Reference]	1 [Reference]
Male	2.7 (0.9-8.0)	1.0 (0.6-1.7)
**Race/ethnicity**		
Non-Hispanic white	1 [Reference]	1 [Reference]
Non-Hispanic black	0.8 (0.3-2.0)	1.8 (1.0-3.5)
Mexican American	0.4 (0.2-1.8)	0.7 (0.3-1.5)
**Education**		
Some college or greater	1 [Reference]	1 [Reference]
Less than high school	4.5 (1.8-11.2)	1.6 (0.8-3.0)
High school graduate or GED	1.4 (0.5-3.4)	1.9 (1.0-3.6)
**Health insurance coverage**		
Absent	1 [Reference]	1 [Reference]
Present	0.4 (0.1-1.8)	0.5 (0.2-1.2)
**Overweight or obese^c^**		
**Sex**		
Female	1 [Reference]	1 [Reference]
Male	1.0 (0.4-2.3)	0.8 (0.4-1.4)
**Race/ethnicity**		
Non-Hispanic white	1 [Reference]	1 [Reference]
Non-Hispanic black	0.7 (0.3-1.8)	1.8 (0.9-3.4)
Mexican American	0.4 (0.2-1.0)	1.4 (0.7-2.9)
**Education**		
Some college or greater	1 [Reference]	1 [Reference]
Less than high school	0.4 (0.1-1.3)	1.9 (1.0-3.8)
High school graduate or GED	0.3 (0.1-1.0)	1.6 (0.8-3.3)
**Health insurance coverage**		
Absent	1 [Reference]	1 [Reference]
Present	1.0 (0.2-5.5)	2.4 (1.0-5.8)

Abbreviations: LED, lower extremity disease; AOR, adjusted odds ratio; CI, confidence interval; GED, general educational development certificate; HDL, high-density lipoprotein.

a All models adjusted for age.

b Confidence intervals may begin with 1 because of rounding.

c See Methods section for definitions.
